# Histopathological and radiographical evaluation of caprine demineralized bone matrix in a critical ulnar defect in a rabbit model

**DOI:** 10.1186/s13018-022-03454-1

**Published:** 2022-12-23

**Authors:** Olawale Alimi Alimi, Adamu Abdul Abubakar, Abubakar Sadiq Yakubu, Sani Abdullahi Shehu, Salman Zubairu Abdulkadir

**Affiliations:** 1 Department of Veterinary Surgery and Radiology, University of Ilorin, Ilorin, Nigeria; 2 Department of Veterinary Surgery and Radiology, Usmanu Danfodiyo University, Sokoto, Nigeria; 3 Department of Veterinary Anatomy, Usmanu Danfodiyo University, Sokoto, Nigeria

**Keywords:** Rabbit model, Bone grafting, Bone xenograft, Demineralized bone matrix, Fracture healing, Osteoinduction, Osteoconduction

## Abstract

**Background:**

Caprine species satisfy the conditions of an ideal donor animal when compared to bovine species that has been extensively studied and commercialized for bone xenograft. Histopathological and radiological evaluations of caprine demineralized bone matrix (CDBM) were therefore carried out for fracture healing properties for its possible use in bone grafting procedures.

**Materials and methods:**

Twenty-four rabbits were used for this study and were divided randomly into three groups of eight (*n* = 8) rabbits each. Critical bone defect was created on the ulnar diaphysis under xylazine-ketamine anaesthesia for autogenous bone graft (ABG) group, CDBM group and the last group was left unfilled as negative control (NC). Immediate post-grafting radiograph was taken and repeated on days 14, 28, 42 and 56 to monitor the evidence of radiographic healing. The animals were euthanized on day 56 and defect sites were harvested for histopathology.

**Results:**

There was a progressive evidence of radiographic healing and bone formation in all the groups with significance difference (*P* = 0.0064). When compared with ABG, NC differ significantly (*P* < 0.0001) whereas the CDBM did not differ significantly (*P* = 0.6765). The histopathology sections of ABG and CDBM showed normal bone tissue while the NC section was predominated by fibrous connective tissue. There was therefore an overall significant difference (*P* = 0.0001) in which CDBM did not differ from ABG (*P* = 0.2946) while NC did (*P* = 0.0005).

**Conclusion:**

The ABG and CDBM groups showed a similar healing effect in the critical bone defect. Therefore, CDBM could be used as an effective alternative to ABG in orthopaedics to circumvent the limitations and complications associated with it.

**Level of evidence:**

Not applicable.

## Introduction

Bone grafting is indicated in many fractures managed by open reduction and internal fixation, and therefore, at least one donor site for cancellous bone grafting ought to be prepared [1] as they are rich in osteoblasts and osteocytes that will enhance bone healing at the fracture site [2]. The most common site of harvest of autologous bone graft is the iliac crest [3–7]. Other sites include the proximal part of the tibia, distal aspect of the radius and tibia and the greater trochanter of the femur [5, 8, 9]. Proximity of the bone graft site to vascular and neurologic structures accounts for many of the most serious complications of bone graft surgery [4]. Reported major complications include pseudoaneurysm of the pelvic vasculature, arteriovenous fistula, massive blood loss [4, 10], pelvic instability presenting as low back pain, donor site pain, increased blood loss, increased operative time and the potential for donor site infection [10, 11]. Additionally and most importantly, there exists an inherently limited supply of graft [11].

Autogenous bone is the golden standard of bone graft material in orthopaedic surgery [12]. Bone allograft is another alternative harvested from human cadaveric donors [8], but could only be used as a scaffold as its osteogenicity and osteoinductivity are greatly reduced during process of sterilization though it still proffers mechanical support [9, 13, 14]. Allografts could be made available from an unlimited source, reducing the operative time and averting donor site morbidity [9], but poses the risk of transmitting infections especially viral, though low but with moderate to high severity when it occurs [15], immune and inflammatory responses post-implantation [16, 17] and increased demand for bone reconstruction had led to a relative deficiency in allogeneic donors as well [14]. The disadvantages of both autograft and allograft intensified the research into finding other bone substitutes like xenograft [14, 18].

There has been a long list of possible xenograft donors to human; however, there are criteria to be met which include compatibility of the anatomy and physiology of the intended organ [19]. Others are the absence of zoonotic disease tendency, the species should be cheap to feed and breed, resistant to human diseases (especially viral), has short gestation with multiple litter per birth, present no immunologic barriers to transplantation to humans and lastly, has little or no ethical controversies [19].

The bovine bone and its derivatives such as hydroxyapatite, collagen, demineralized bone matrix (DBM) and de-proteinized bone have been used in previous studies [20, 21], and focus was made on differences in the stimulating effect of the core bone DBM in bone healing. The xenogenic bovine DBM has previously been compared with fresh autogenous cortical bone in rabbit model and the results were promising [22]. However, bovine source of bone xenograft does not satisfy the conditions of ideal donor animal as opposed to small ruminants based on long gestation, high cost of keeping and monoparity [19]. Therefore, small ruminants like caprine could be considered due to their short gestation, pluriparity and low cost of keeping against bovine if to be kept for the purpose of harvesting grafts based on conditions stated by Levy [19]. Furthermore, Banerjee et al. [23] had compared the microanatomy structures of the cancellous bone of goat and human mandibles and found out that there is similarity and that goat’s mandible could be used for implant experiment. In the current study, we therefore evaluated caprine demineralized bone matrix in an ulnar critical bone defect for osteoconductive and osteoinductive properties in rabbit model.

## Materials and method

### Animals

Twenty-four mature male Nigerian local rabbits with body weight 2.5 ± 0.5 kg were acquired for the purpose of this study and were allowed to acclimatize for two weeks. The skeletal maturity of the rabbits was confirmed by radiographical evidence of closure of distal femur, proximal tibia and complete fusion of olecranon process. They were randomly divided into three groups: ABG, NC and CDBM group. Ethical approval was sought from the Faculty Animal Research Ethics Committee of the Faculty of Veterinary Medicine, Usmanu Danfodiyo University, Sokoto, with the reference number UDUS/FAREC/2019/AUP-R0-5.

### Preparation of CDBM

Long bones of goat were collected from the slaughterhouse for the preparation of DBM. All soft tissues were removed and the bones were cut into 1 cm pieces with a saw. The pieces were decalcified in 0.6 mol/L HCl (BIC Chemicals, Maharashtra, India) at 4 °C for 8 days as described by Monazzah et al. [24]. After eight days, the DBM were washed with distilled water, packed and stored at 4 °C till use.

### Critical bone defect model and bone graft implantation

Anaesthesia was achieved using an intramuscular injection of ketamine (Ketalar®, International Limited, Lagos, Nigeria) (100 mg/kg, IM) and xylazine hydrochloride (XYL-M2®, VMD, Arendonk, Belgium) (50 mg/kg, IM). The left forelimbs were clipped and prepared aseptically for surgery using chlorhexidine (Purit®, Saro Lifecare Ltd., Lagos, Nigeria), methylated spirit (La Onyz®, Samstella Nigeria Limited, Abule Oba, Nigeria) and povidone iodine (Wosan®, Jawa International Limited, Lagos, Nigeria). The limbs were draped with sterile draping materials.

A skin incision was made over the ulnar bone, and the bone was exposed by retracting the extensor muscles. A non-union bone model, an osteoperiosteal segmental defect of approximately 1 cm was created on the ulna bone according to standard procedure previously described [12, 24, 25].

In ABG, the defected areas were filled with same ostectomized bone to serve as autologous graft, the defects were left unfilled in NC and the defect was filled with same length of caprine DBM in CDBM. The grafts were secured in place with the surrounding muscles and sutured using simple continuous suture pattern with chromic catgut (Agary Pharmceuticals, Jiangsu, China) size 3/0. The skin was sutured using interrupted horizontal suture pattern with nylon (Agary Pharmceuticals, Jiangsu, China) (size 2/0).

Post-operative analgesia and antibiosis were achieved using diclofenac sodium (Yanzhou Xier Kangtai Pharmaceutical, Yanzhou, China) at 3 mg/kg intramuscularly for three days and Penicillin–Streptomycin (Hebei Hope Harmony Pharmaceutical Co. Ltd., China) at 25 mg/kg intramuscularly for five days.

### Radiological evaluations

For radiological evaluation, radiographs of the defected areas were taken immediately post-surgery to ascertain the defect and correct filling of the defected area. Subsequently, the radiograph was taken at 14th, 28th, 42nd and 56th post-operative days. Radiological criteria evaluated were bone formation, bone union, and remodelling. The radiological findings were scored with modification according to radiological scoring earlier reported [17, 26, 27]. Briefly, the scoring was done based on evidence of bone formation, presence of fracture line and bone remodelling. For bone formation, no evidence of bone formation was scored 0 while 25%, 50%, 75% and 100% bone formed occupying the defect were scored 1, 2, 3 and 4, respectively. The union was considered based on the presence of full, partial and absence of fracture line and were scored 0, 2 and 4, respectively. Lastly, the bone remodelling was scored as no remodelling (0), remodelling of the intramedullary channel (2) and full remodelling of the cortex (4). The results were presented as the sum of all the radiographic scores. All radiographs were taken under light sedation (xylazine, 25 mg/kg) to prevent animal movement during exposure. The radiographs were evaluated by two independent radiologists that were blinded with the experimental design.

### Histopathological evaluations

On the 56th post-operative day, the experimental animals were euthanized by means of cervical dislocation after anaesthesia for histopathological evaluations [28]. The operated limbs were harvested and the bones were dissected free of soft tissue. The defect sites and the graft were ostectomized and fixed in 10% buffered formalin. The formalin-fixed bone samples were transferred into 0.6 mol/L HCl for eight days at 4 °C for decalcification [17]. Standard histologic processing and Haematoxylin and Eosin staining technique was carried out as stated by Bigham-Sadegh and Oryan [12]. The slides were examined under a light microscope and photomicrographs were taken. All histopathological sections were evaluated and scored using Emery histopathological formation criteria as reported by Bigham et al. [29]. In this criterion, an empty space without tissue was scored 0 while the presence of fibrous tissue only was scored 1, more fibrous tissue than fibrocartilage was scored 2, more fibrocartilage than fibrous tissue was scored 3, fibrocartilage only was scored 4, more fibrocartilage than bone was scored 5, more bone than fibrocartilage was scored 6 and bone only was scored 7. The histological evaluation was conducted by two independent histopathologists that were blinded to the experimental design.

### Statistical analysis

All retrieved data from radiological scoring were analysed using a two-way ANOVA repeated measures mixed model approach with rank transformation of the scores prior to analysis to stabilize the variance. The histopathological data were analysed using Kruskal–Wallis, nonparametric ANOVA and significance level was determined when *P* value is < 0.05, pairwise group comparisons was performed using Mann–Whitney *U* Test (InVivoStat version 4.0.2).

## Results

All animals recovered uneventfully from anaesthesia and there was no intra-surgical and post-surgical complication.

### Radiographic result

**Fig. 1:**
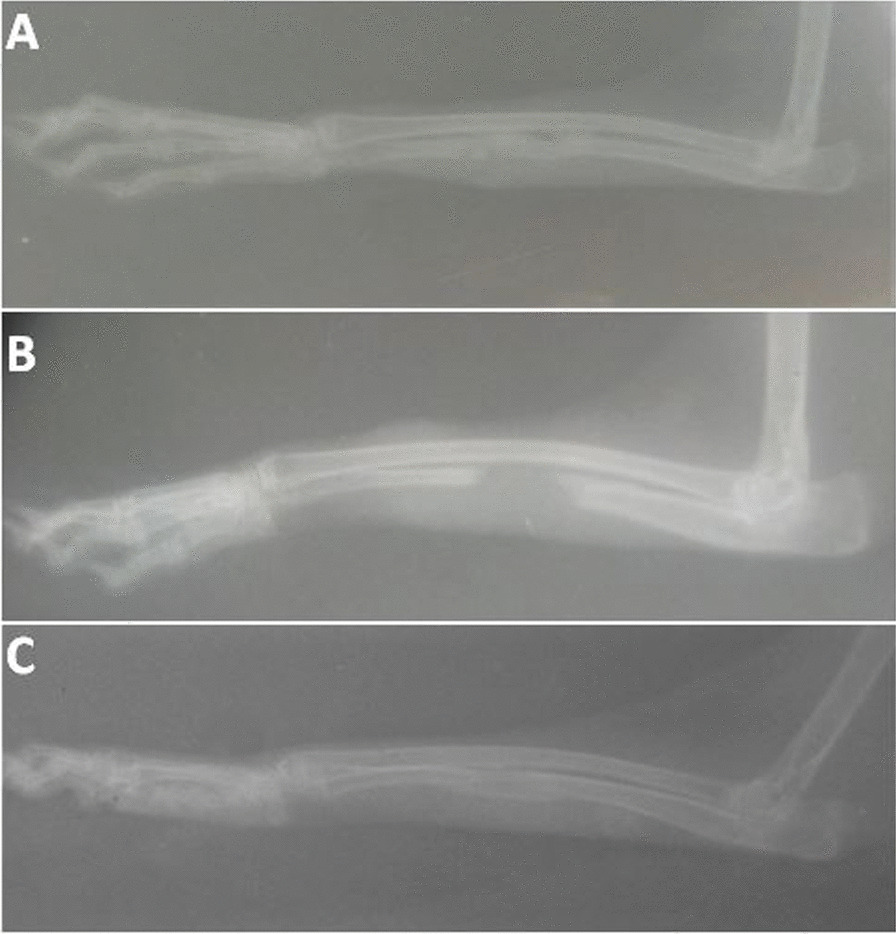
Radiographic appearance of the defects on day 28

**Fig. 2:**
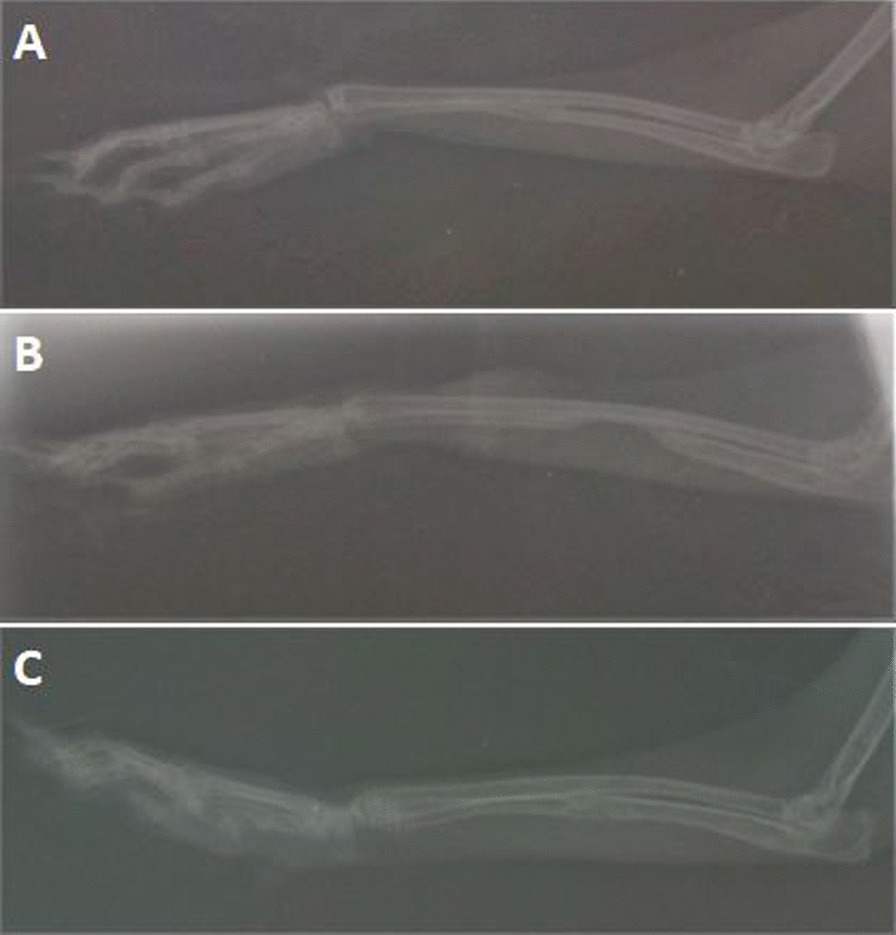
Radiographic appearance of the defects on day 56

There was a progressive bone formation and union from day 14 through day 42 when remodelling was observed to have started and to day 56 when the union and remodelling had completed in ABG and CDBM while there was no substantial radiographic evidence of bone union in NC. The radiographs of days 28 and 56 are presented in Figs. 1 and 2 representing the middle and the end of the observation, respectively. There was overall significance difference (*P* = 0.0064) across the groups through days 14 to 56. There was, however, no pairwise significance difference (*P* = 0.6765) between groups ABG and CDBM, while a marked pairwise significance (*P* < 0.0001) exists between ABG and NC; NC and CDBM. Table 1 shows the radiographic score result observed in the groups across the days.

**Table 1:** Radiographical scores expressed in median (minimum–maximum) (*n* = 8)

Post-surgery days	Group			*P*-value
	ABG	NC	CDBM	
Day 14	3.00 (0.00–3.50)	0.00 (0.00–1.50)*	3.50 (1.50–5.00)	< 0.0001
Day 28	8.50 (5.50–9.00)	3.25 (0.00–6.00)*	9.00 (3.00–9.50)	0.0002
Day 42	10.00 (7.00–10.50)	4.25 (0.00–10.00)*	10.75 (9.00–12.00)	< 0.0001
Day 56	11.00 (9.00–12.00)	6.00 (0.00–9.00)*	12.00 (8.50–12.00)	< 0.0001

The median bearing (*) differ significantly from the ABG

### Histopathology result

**Fig. 3:**
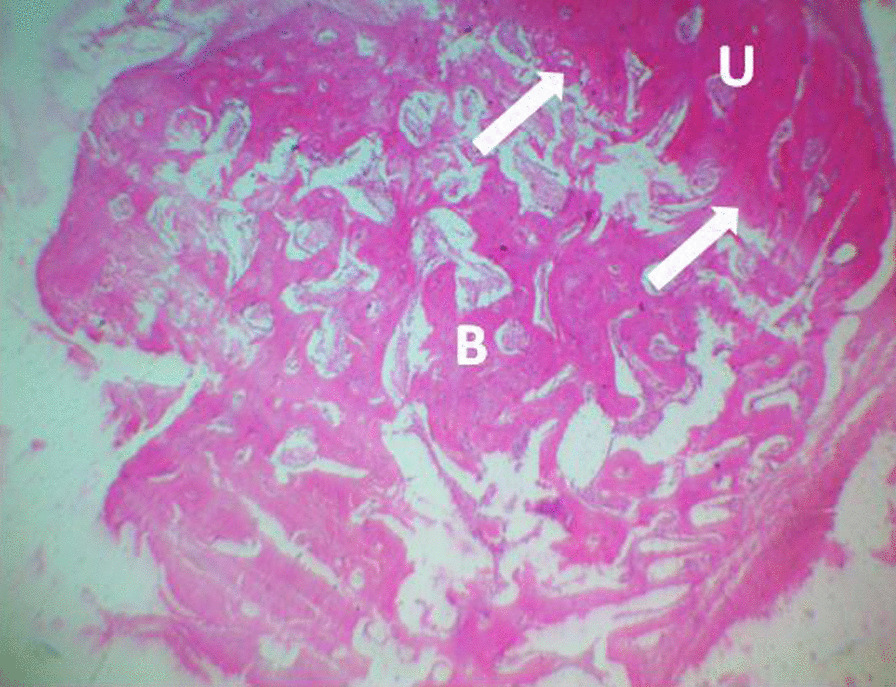
Histopathological photomicrograph of defect site (ABG); the defect is filled with normal bone tissue and has united with the unaffected part. Bone tissue at the defect site (B), point of union (white arrow) and unaffected bone (U). H&E stain × 100

**Fig. 4:**
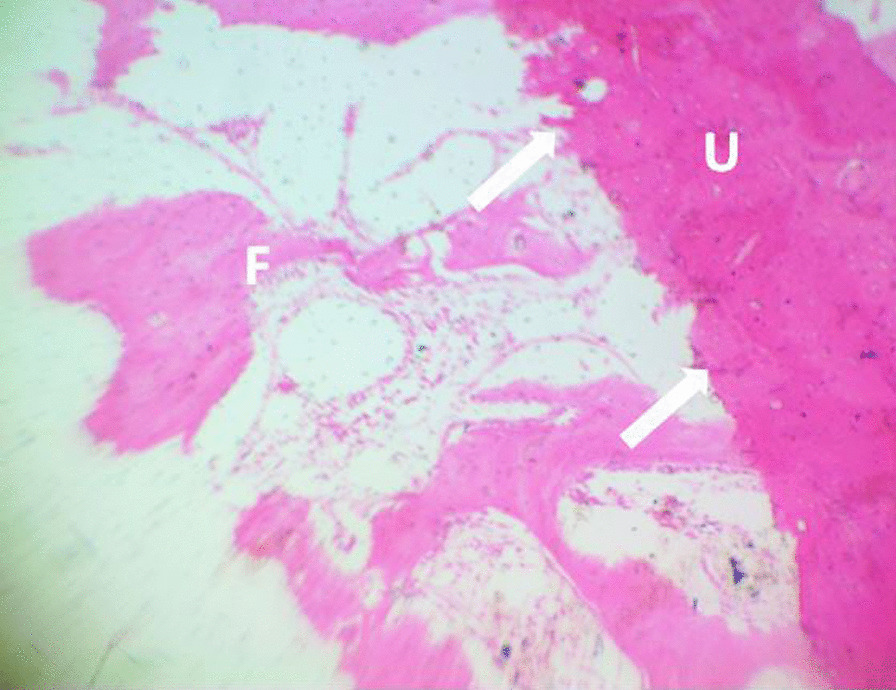
Histopathological photomicrograph of defect site (NC); the defect is filled with fibrous tissue. Fibrous tissue at the defect site (F), point of union (white arrow) and unaffected bone (U). H&E stain × 100

**Fig. 5:**
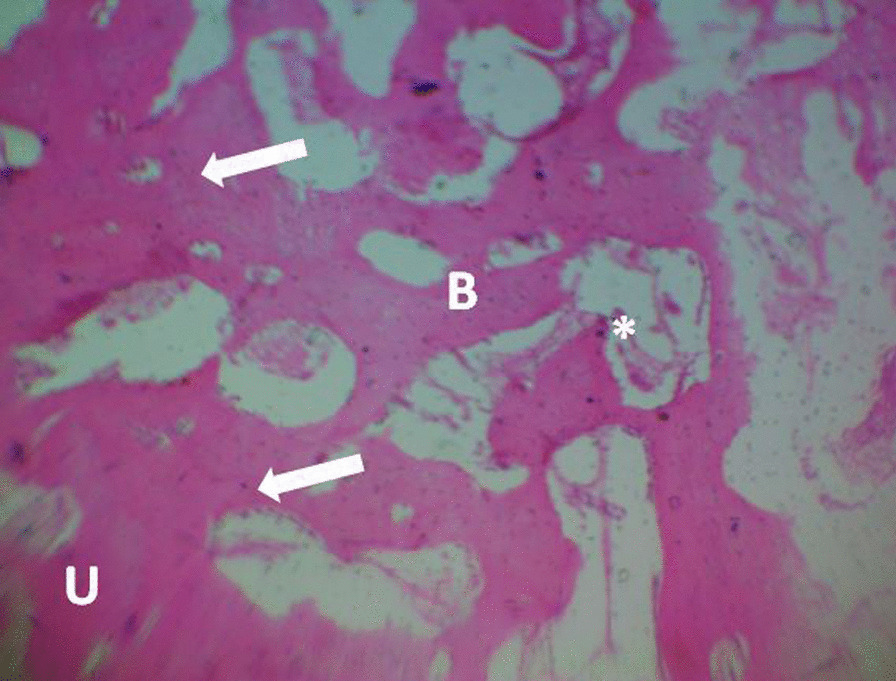
Histopathological photomicrograph of defect site (CDBM); the defect is filled with normal bone tissue and has united with the unaffected part. Bone tissue at the defect site (B), point of union (white arrow), unaffected bone (U) and CDBM remnant (*). H&E stain × 100

The histopathology slides were viewed using Olympus light microscope at X400 magnification and the photomicrographs were taken. The micrographs are shown in Figs. 3, 4 and 5. The histopathology scoring is presented in Table 2. The histopathologic sections in ABG and CDBM showed normal bone histology, whereas the sections in NC are predominated by fibrous connective tissue. There was an overall significant difference (*P* = 0.0001) among the three groups while NC differ significantly (*P* = 0.0005) from the ABG and CDBM did not (*P* = 0.2946).

**Table 2:** Histopathologic scores expressed in median (minimum–maximum) (*n* = 8)

Group			*P*-value
ABG	NC	CDBM	
7.00 (6.00–7.00)	2.50 (3.00–5.0)*	7.00 (6.00–7.00)	0.0001

The median bearing (*) differ significantly from the ABG

## Discussion

Bone autograft being considered the golden standard [12, 14, 24], it was therefore used as the positive control (standard) with which the test was compared. The test was the caprine DBM and the negative control was left empty or untreated with bone graft. There have been reports of the use of bovine DBM and bovine bone xenograft in animal models in bone defects [22, 24, 30]. According to Levy [19], the bovine did not satisfy the conditions of an ideal xenograft donor because it poses the risk of xenosis, not easy and cheap to breed and uniparous.

There was a significant radiographic evidence of progressive bone healing from day 14 through day 56 in the three groups (*P* = 0.0018). A marked significant difference (*P* < 0.0001) was observed between ABG and NC while there was no statistically significant difference (*P* = 0.2859) between ABG and CDBM. This finding showed that the caprine DBM has as good radiographic healing potential as the autologous bone graft. This finding is consistent with the findings of Monazzah et al. [24], who compared bovine DBM with an autologous bone graft and untreated bovine bone in a rat radial defect model and find out that the radiographic healing of bovine DBM was as good as the autologous graft. Also, the study of Bigham et al. [31] and Bigham et al. [29] on bovine DBM and demineralized bovine foetal growth plate in rabbit and rat models, respectively, also showed a non-significant result when compared with the autograft. This result is largely due to the fact that the DBM has many growth factors such as bone morphogenetic proteins (BMPs), insulin-like growth factor, transforming growth factor beta and fibroblast growth factor that are attached to the extracellular collagen matrix and provide the osteoinductive properties. These proteins promote the differentiation of the mesenchymal cells to chondroblastic cells, and new bone formation occurred by means of endochondral osteogenesis [14, 22, 24, 29]. Furthermore, DBM is also porous and provide a three-dimensional scaffold because the fibrous collagen structure of original tissues remains [32]. This justified the reason for its osteoconductive properties and enhancement of cell adhesion and proliferation [15, 33, 34].

An overall significant difference (*P* = 0.0001) was observed in the histopathological score within the groups, significant difference (*P* = 0.0005) was observed when NC was compared with the ABG and there was no significant difference (*P* = 0.2946) between ABG and CDBM. This result showed that histopathologically, insignificant difference between the CDBM and the ABG means they both have similar bone defect healing properties within the period of 60 days used for this study. This result was consistent with that of Monazzah et al. [24] when they compare bovine bone DBM and an autograft in radial defect in rat model. The histopathology of the defect sites were filled with bone tissue in both the test and positive control groups. The test group, however, have osteoclastic and osteoblastic cells signifying an active remodelling process. This result was also consistent with the findings of Bigham et al. [31] in their comparison of bovine DBM and bovine foetal growth plate with the gold standard in radial defect in rabbit. A study where they also found out that there was no significant difference between the histopathology results of the autograft and bovine DBM in a radial bone defect of a rabbit model. Similar significant result and filling of defect site was observed by Bigham-Sadegh et al. [35] when they compare healing pattern of cortical autograft, calf foetal DBM and commercial DBM in tibial defect in dogs. This result was also not different from that reported on the comparison of bovine DBM and autologous cortical bone graft in a radial defect of rabbit [22].

It can be concluded that the CDBM showed the same healing effect as the autologous bone in the critical bone defect. Therefore, CDBM could be a promising alternative to ABG in orthopaedics to circumvent the limitations and complications associated with it. However, there is a need for further studies to compare the ultrastructure of CDBM with human and other animal bones and establish the immunologic response of the recipient to CDBM. The limitation of this study lies in our inability to use a more detailed imaging and histology techniques to prove further our findings, this is due to their unavailability owing to lack of funding.

## Data Availability

All the data is available on genuine request.

## Abbreviations

ABG: Autologous bone graft group
CDBM: Caprine demineralized bone matrix group
NC: Negative (unfilled) control group

## References

[1] Penwick RC, Newton CD, Nunamaker DM (1985). Preoperative patient preparation. Textbook of small animal orthopaedics.

[2] Nandi SK, Roy S, Mukherjee P (2010). Orthopaedic applications of bone graft & graft substitutes: a review. Indian J Med Res.

[3] Arrington ED, Smith WJ, Chambers HG (1996). Complications of iliac crest bone graft harvesting. Clin Orthop Relat Res.

[4] Goulet JA, Senunas LE, DeSilva GL, Greenfield ML (1997). Autogenous iliac crest bone graft. Complications and functional assessment. Clin Orthop Relat Res.

[5] Myeroff C, Archdeacon M (2011). Autogenous bone graft: donor sites and techniques. J Bone Jt Surg Ser A.

[6] Owoola A, Odunubi O, Yinusa W, Unegbu M (2010). Proximal tibial metaphysis: its reliability as a donor site for grafting. West Afr J Med.

[7] Salawu ON, Babalola OM, Ahmed BA (2017). Comparative study of proximal tibia and iliac crest bone graft donor sites in treatment of orthopaedic pathologies. Malays Orthop J.

[8] Griffin KS, Davis KM, McKinley TO (2015). Evolution of bone grafting: bone grafts and tissue engineering strategies for vascularized bone regeneration. Clin Rev Bone Miner Metab.

[9] O’Malley MJ, Sayres SC, Saleem O (2014). Morbidity and complications following percutaneous calcaneal autograft bone harvest. Foot Ankle Int.

[10] Khan SN, Cammisa FP, Sandhu HS (2005). The biology of bone grafting. J Am Acad Orthop Surg.

[11] Roberts TT, Rosenbaum AJ (2012). Bone grafts, bone substitutes and orthobiologics. The bridge between basic science and clinical advancements in fracture healing. Organogenesis.

[12] Bigham-Sadegh A, Oryan A (2015). Selection of animal models for pre-clinical strategies in evaluating the fracture healing, bone graft substitutes and bone tissue regeneration and engineering. Connect Tissue Res.

[13] Cross DJ, DiDomenico LA (2019). Calcaneal bone graft procedures: an analysis of postsurgical complications. J Foot Ankle Surg.

[14] Sohn HS, Oh JK (2019). Review of bone graft and bone substitutes with an emphasis on fracture surgeries. Biomater Res.

[15] Flynn J, Markel G (2012). Characterization of the inflammatory response to four commercial bone graft substitutes using a murine biocompatibility model. J Inflamm Res.

[16] Bauer TW, Muschler GF (2000). Bone graft materials: an overview of the basic science. Clin Orthop Relat Res.

[17] Bigham-Sadegh A, Mirshokraei P, Karimi I (2012). Effects of adipose tissue stem cell concurrent with greater omentum on experimental long-bone healing in dog. Connect Tissue Res.

[18] Greenwald AS, Boden SD, Goldberg VM (2001). Bone-graft substitutes: facts, fictions, and applications. J Bone Jt Surg Am.

[19] Levy MF (2000). Animal organs for human transplantation: how close are we?. Proc (Bayl Univ Med Cent).

[20] Oryan A, Monazzah S, Bigham-Sadegh A (2015). Bone injury and fracture healing biology. Biomed Environ Sci.

[21] Oryan A, Alidadi S, Moshiri A, Maffulli N (2014). Bone regenerative medicine: classic options, novel strategies, and future directions. J Orthop Surg Res.

[22] Bigham AS, Dehghani SN, Shafiei Z, TorabiNezhad S (2008). Xenogenic demineralized bone matrix and fresh autogenous cortical bone effects on experimental bone healing: radiological, histopathological and biomechanical evaluation. J Orthop Traumatol.

[23] Banerjee S, Chakraborty A, Pal T (2014). A micro-anatomical comparison of goat jaw cancellous bone with human mandible: histomorphometric study for implant dentistry. J Int Clin Dent Res Organ.

[24] Monazzah S, Oryan A, Bigham-Sadegh A, Meimandi-Parizi A (2017). Application of bovine bone versus bovine DBM graft on bone healing of radial defect in rat. Comp Clin Path.

[25] Arpağ OF, Damlar I, Altan A (2018). To what extent does hyaluronic acid affect healing of xenografts? A histomorphometric study in a rabbit model. J Appl Oral Sci.

[26] Korkmaz M, Oztürk H, Bulut O (2005). The effect of definitive continuous distraction employed with the Ilizarov type external fixation system on fracture healing: an experimental rabbit model. Acta Orthop Traumatol Turc.

[27] Ajai S, Sabir A, Mahdi AA, Srivastava RN (2013). Evaluation of serum alkaline phosphatase as a biomarker of healing process progression of simple diaphyseal fractures in adult patients. Int Res J Biol Sci.

[28] Hickman DL, Johnson SW (2011). Evaluation of the aesthetics of physical methods of euthanasia of anesthetized rats. J Am Assoc Lab Anim Sci.

[29] Bigham AS, Shadkhast M, BighamSadegh A (2011). Evaluation of osteoinduction properties of the demineralized bovine foetal growth plate powder as a new xenogenic biomaterial in rat. Res Vet Sci.

[30] MeimandiParizi A, Oryan A, Shafiei-Sarvestani Z, Bigham AS (2012). Human platelet rich plasma plus Persian Gulf coral effects on experimental bone healing in rabbit model: radiological, histological, macroscopical and biomechanical evaluation. J Mater Sci Mater Med.

[31] Bigham AS, Dehghani SN, Shafiei Z, Nezhad ST (2009). Experimental bone defect healing with xenogenic demineralized bone matrix and bovine fetal growth plate as a new xenograft: radiological, histopathological and biomechanical evaluation. Cell Tissue Bank.

[32] Lane JM (2005). Bone morphogenic protein science and studies. J Orthop Trauma.

[33] Oakes DA, Lee CC, Lieberman JR (2003). An evaluation of human demineralized bone matrices in a rat femoral defect model. Clin Orthop Relat Res.

[34] Zambuzzi WF, de Oliveira RC, Pereira FL (2006). Rat subcutaneous tissue response to macrogranular porous anorganic bovine bone graft. Braz Dent J.

[35] Bigham-Sadegh A, Karimi I, Alebouye M (2013). Evaluation of bone healing in canine tibial defects filled with cortical autograft, commercial-DBM, calf fetal DBM, omentum and omentum-calf fetal DBM. J Vet Sci.

